# Past, Current, and Future Strategies to Target ERG Fusion-Positive Prostate Cancer

**DOI:** 10.3390/cancers14051118

**Published:** 2022-02-22

**Authors:** Francesca Lorenzin, Francesca Demichelis

**Affiliations:** 1 Department of Cellular, Computational and Integrative Biology, CIBIO, University of Trento, 38123 Trento, Italy; 2 The HRH Prince Alwaleed Bin Talal Bin Abdulaziz Al-Saud Institute for Computational Biomedicine, Weill Cornell Medical College, New York, NY 10021, USA; 3 The Caryl and Israel Englander Institute for Precision Medicine, Weill Cornell Medicine, New York, NY 10021, USA

**Keywords:** ERG, prostate cancer, transcription factor, therapeutic strategies

## Abstract

**Simple Summary:**

In addition to its role in development and in the vascular and hematopoietic systems, ERG plays a central role in prostate cancer. Approximately 40–50% of prostate cancer cases are characterized by *ERG* gene fusions, which lead to ERG overexpression. Importantly, inhibition of ERG activity in prostate cancer cells decreases their viability. Therefore, inhibiting ERG might represent an important step to improve treatment efficacy for patients with ERG-positive prostate tumors. Here, we summarize the attempts made over the past years to repress ERG activity, the current use of ERG fusion detection and the strategies that might be utilized in the future to treat ERG fusion-positive tumors.

**Abstract:**

The ETS family member ERG is a transcription factor with physiological roles during development and in the vascular and hematopoietic systems. ERG oncogenic activity characterizes several malignancies, including Ewing’s sarcoma, leukemia and prostate cancer (PCa). In PCa, *ERG* rearrangements with androgen-regulated genes—mostly *TMPRSS2*—characterize a large subset of patients across disease progression and result in androgen receptor (AR)-mediated overexpression of ERG in the prostate cells. Importantly, PCa cells overexpressing ERG are dependent on ERG activity for survival, further highlighting its therapeutic potential. Here, we review the current understanding of the role of ERG and its partners in PCa. We discuss the strategies developed in recent years to inhibit ERG activity, the current therapeutic utility of ERG fusion detection in PCa patients, and the possible future approaches to target ERG fusion-positive tumors.

## 1. Introduction

The E-26 transformation-specific (ETS) family of transcription factors includes 28 members encoded by the human genome [1,2]. ETS members are defined by a conserved 85 amino acid-long ETS domain that binds DNA over a region of 15–20 base pairs with a core 5′-GGA(A/T)-3′ sequence [3]. Limited homology is instead observed in the rest of the protein structure, with some members—including ERG and FLI1—presenting a pointed (PTN) domain important for protein–protein interactions, while others—including members of the PEA3 subfamily such as ETV1, ETV4 and ETV5—characterized by a transactivation domain in the *N*-terminal [4]. Among the ETS factors are both transcriptional activators and repressors, which control a plethora of processes, including differentiation, proliferation, apoptosis, tissue remodeling and angiogenesis [5]. Their expression and activity are deregulated in many tumor types, with some members having a tumor-suppressive role, while others promote and sustain oncogenic behaviors [6]. Given the high homology of the DNA binding domain, questions have arisen about how ETS proteins can behave so differently and regulate such a diverse collection of processes [7]. Several factors can affect specificity: functional specialization is partially explained by the tissue-specific expression and the divergent expression patterns observed for some ETS members, while others are ubiquitously expressed [8,9]. Phylogenetic and chromatin immunoprecipitation followed by sequencing (ChIP-seq) studies showed that small differences in the protein sequence of the ETS domain alter DNA sequence specificity—with ETS member’s preference for different flanking sequences to the GGA(A/T) core—and affect in vivo occupancy [10]. Interaction and cooperative binding with other transcription factors shape binding specificity and can be modulated by domains that are different among the ETS members. These domains can also be post-translationally modified, adding a further level of control that could affect specificity. Characterizing all these aspects is fundamental to understand the activity of ETS members in disease and for the development of therapeutic strategies with high specificity and efficacy.

**Figure 1:**
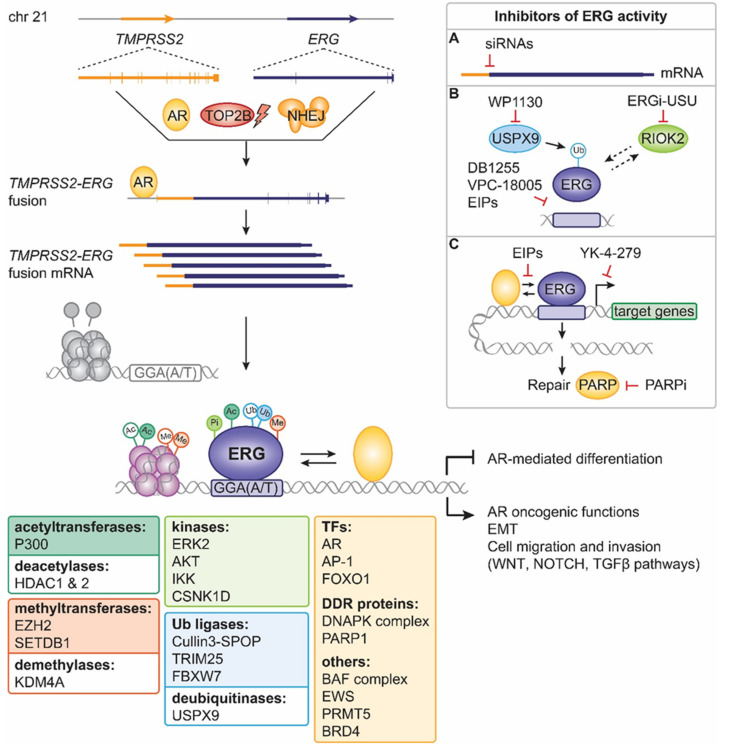
Schematic of the key events and players affecting ERG levels and activity in PCa. The regulatory region of the androgen-regulated gene *TMPRSS2* is fused to the coding sequence of *ERG* due to the activity of AR, which recruits TOP2B and can cause DNA double strand breaks that are repaired via NHEJ. Under the control of AR, the *TMPRSS2-ERG* fusion transcript is produced at high levels and translated into wild-type ERG or *N*-terminally truncated ERG that retains all important functional domains. ERG binds to DNA at specific sequences and is targeted by several post-translational modifications (Ac = acetylation, dark green; Me = methylation, orange; Ub = ubiquitination, blue; and Pi = phosphorylation; light green) controlled by diverse proteins (dark green, orange, blue and light green boxes). Together with its interactors (yellow box), ERG regulates target gene expression and processes that positively affect prostate tumorigenesis and prostate cancer maintenance. The upper right inset summarizes the inhibitors of ERG activity developed so far, which: (**A**) affect *TMPRSS2-ERG* mRNA stability (siRNA targeting the fusion breakpoint); (**B**) decrease ERG protein stability and inhibit DNA binding; (**C**) alter ERG interaction with cofactors and transcriptional activity or exacerbate DNA damage. EIPs = ERG inhibitory peptides, EMT = epithelial-to-mesenchymal transition, TFs = transcription factors, DDR = DNA damage response.

Among the ETS members, ERG (ETS-related gene) plays important roles in normal physiology and tumorigenesis, with its overexpression detected in several tumor types, including Ewing’s sarcoma, hematological malignancies and prostate cancer (PCa). The role and activity of ERG have been extensively studied in PCa—one of the most common malignancies diagnosed among men and a major cause of death [11]—where ERG mutations are found in about half of patients with primary and advanced disease [12,13]. Yet, the oncogenic implications of ERG overexpression and its clinical utility are still debated. This review discusses the role of ERG in normal tissue and in PCa, with focus on its partners in activity and the past, present and future strategies to target ERG-positive PCa (Figure 1).

## 2. ERG Functions in Development and Normal Physiology

ERG is expressed during embryogenesis in mesodermal tissues, predominantly in the developing endothelium and cartilage, and neural crests. While its expression decreases during development, it remains highly expressed in the endothelial cells of most adult tissues and in the hematopoietic system [14,15,16,17,18]. ERG knockout in mice is embryonically lethal due to defects in the cardiac and vascular development related to the incapacity of endothelial cells to undergo endothelial-to-mesenchymal transition in order to migrate and to form new blood vessels [19,20]. Moreover, the depletion of ERG in murine embryonic stem cells decreases their capacity to differentiate into endothelial cells [21]. In adults, ERG is involved in vascular homeostasis and angiogenesis by regulating pathways affecting vascular integrity, endothelial permeability and survival [22,23,24,25]. It controls the expression of endothelial cell-specific genes (e.g., VE-cadherin, claudin5, vWF, endoglin, eNOS) and regulates the WNT/b-catenin pathway, promoting vascular stability and growth [20,26,27]. By controlling cytoskeleton dynamics, ERG regulates migration of endothelial cells [23]. In resting human umbilical vein endothelial cells (HUVEC), ERG inhibits the binding of NF-κB p65 to the promoter of several pro-inflammatory genes leading to their repression, thus protecting from improper endothelial activation [28].

In the hematopoietic system, ERG regulates programs related to stem cell self-renewal. In vivo studies showed that ERG is required to maintain the hematopoietic stem cell (HSC) compartment and to sustain definitive hematopoiesis by preventing HSC exhaustion [29,30]. ERG is expressed transiently during T-cell specification, while it is silent in mature T-lymphocytes [31]. Ectopic ERG overexpression in T- and B-lymphocytes alters differentiation and promotes the growth of immature precursor cells [32]. Accordingly, rearrangements and/or aberrant expression of ERG are found in acute myeloid leukemia [33,34,35]. Finally, a role in preventing differentiation into hypertrophic cells was also observed for ERG in articular chondrocytes [36,37].

## 3. ERG Interactors and Post-Translational Modifications

Several functional domains are present in the ERG protein. Through the C-terminal ETS domain, ERG binds DNA preferentially at the 5′- ACC(GGAA)GT- 3′ sequence [10]. The regions flanking the ETS domain have inhibitory activity by decreasing the binding affinity to DNA. Although ERG autoinhibition of DNA binding is limited, with only a modest change in binding affinity (about three-fold reduction), other ETS members exhibit stronger autoinhibition (10–30 fold) [38,39]. A pointed (PNT) domain characterizes the central part of ERG, which enables homo- and heterodimerization with other ETS factors [40,41]. However, ERG homodimerization seems to be incompatible with DNA binding and transcriptional regulation [40].

Post-translational modifications and association of ERG with other transcription factors and chromatin-modifying enzymes can affect ERG protein levels and binding to DNA influencing the transcriptional control exerted on its target genes. 

The RAS/MAPK pathway regulates activation of several ETS factors, including ERG [42]. ERK2 phosphorylates ERG at serine 96, 215 and 283. Phosphorylation of serine 283 is exclusively detected in leukemic cells compared to hematopoietic and progenitor stem cells and associates with increased ERG binding to the regulatory elements of specific genes contributing to the malignant phenotype of ERG-driven leukemia [43]. Phosphorylation of serine 96 and 215 is instead observed in prostate cancer cells. An in vitro study showed that phosphorylated serine 215 induces a conformational change in ERG, which primes for the subsequent phosphorylation at serine 96. This second modification decreases the affinity of ERG for Enhancer Of Zeste 2 Polycomb Repressive Complex 2 Subunit (EZH2), the catalytic subunit of the Polycomb Repressive Complex 2 (PRC2) and leads to the loss of EZH2 binding across the ERG cistrome, promoting ERG-mediated transcriptional activation and cell migration [44,45]. Mass spectrometry analysis using the VCaP prostate cancer cell line confirmed the phosphorylation at serine 215 and identified serine 81 as a newly phosphorylated residue in the ERG protein. Treatment of VCaP with several inhibitors showed that ERG serine phosphorylation was dependent on AKT and IKK kinases and mediates *CXCR4* expression, a gene implicated in the interaction between tumor cells and the surrounding microenvironment [46]. Additional studies using prostate cancer models showed phosphorylation of ERG at residues in the *N*-terminal degron by casein kinase I δ, which triggers ubiquitination by the Cullin3-SPOP E3 ubiquitin ligase complex and consequent degradation [47,48]. ERG degradation is also favored by the activity of the E3 ubiquitin ligase TRIM25 that targets the C-terminal degron [49] and FBW7 that ubiquitinates ERG upon priming by DNA damage-trigged phosphorylation [50]. Conversely, USP9X deubiquitinates and stabilizes ERG [51]. Methylation of ERG at lysine 362 (K362) was recently reported as a novel PRC-2-independent function of EZH2. Methylated K362 changes the intra-domain interaction between the ETS domain, where K362 is located, and the C-terminal inhibitory domain increasing accessibility and favoring binding to DNA, with consequent enhanced ERG transcriptional and oncogenic activity [52].

ChIP-seq analyses for several ETS members in prostate cancer cells identified overrepresentation of the AP-1 binding site in regions occupied by ERG, ETV1 and ETV4 [53]. AP-1 transcription factors are dimers of basic region-leucine zipper (bZIP) proteins belonging to the JUN, FOS, MAF and ATF sub-families and play important roles in cellular proliferation, survival, locomotion and tumor biology [54]. Furthermore, in vitro studies showed that the ETS domain is responsible for the interaction between ERG and JUN/FOS heterodimers. This interaction is not dependent on DNA and JUN/FOS have minimal effects on the DNA binding affinity of ERG to ERG-AP-1 composite sites [55]. Concomitant binding of ERG and AP-1 complexes has a synergistic effect on transcriptional activation in luciferase assay [56]. Oppositely to this, the interaction of ERG with FOXO1, a forkhead transcription factor frequently inactivated in prostate cancer, inhibits ERG-mediated transcriptional activation [57].

Interaction between ERG and the transcriptional activator and RNA-binding protein EWS [58,59] has also been described. EWS belongs to the FET family and is often found in Ewing’s sarcoma fused to ETS members, including FLI1 and ERG. Additionally, TLS, another member of the family, is fused with ERG in myeloid leukemia [60]. In prostate cancer cells, EWS is necessary for ERG to induce migration and anchorage-independent growth and acts as a co-activator that is recruited by ERG at its target genes [58].

In leukemia and prostate cancer cells, interaction between ERG and bromodomain containing protein 4 (BRD4) has been observed. BRD4 usually recognizes acetylated histones and recruits pTEFB (CyclinT1/CDK9 complex) to genes promoting transcriptional activation [61]. Full-length ERG and several truncated ERG variants detected in prostate cancer contain in the *N*-terminus a KGGK motif acetylated by the acetyltransferase p300. ERG in conjunction with p300 activity recruits BRD4 at its target genes, leading to their transcriptional activation and supporting leukemia maintenance and prostate cancer cell invasion [62,63]. Transcriptional cooperation between ERG and another histone-modifying enzyme was reported at the *YAP1* gene. Here, ERG leads to decreasing histone 3 lysine 9 trimethylation through recruitment of the demethylase KDM4A [64].

To identify ERG interactors, approaches based on immunoprecipitation followed by mass spectrometry were also employed. Brenner and colleagues discovered the interaction between ERG and proteins involved in DNA repair. Specifically, ERG was shown to interact with and recruit to its target genes components of the DNA-dependent protein kinase (DNA-PK) complex, including the catalytic subunit and its interacting proteins, Ku70 and Ku80, and PARP1. Moreover, inhibition of the enzymatic activity of these proteins hampered ERG-mediated transcriptional regulation with a consequent reduction in the capacity of prostate cancer cells overexpressing ERG to invade [65]. In a similar fashion, by using immunoprecipitation followed by stable isotope labelling with amino acids in cell culture (SILAC)-based proteomic mass spectrometry, the Kadoch’s laboratory revealed the direct interaction of ERG with components of the mammalian SWI/SNF (BAF) complex [59]. They further showed that ERG recruits BAF complexes to sites enriched for ETS motifs and that BAF complex activity is required for global ERG chromatin occupancy and target gene regulation. This interdependency for chromatin targeting between ERG and BAF complexes drives basal-to-luminal transition in prostate organoids suggesting its key role in promoting prostate tumorigenesis.

ERG also interacts with the androgen receptor (AR), EZH2, PRMT5 and the histone deacetylases (HDACs) 1 and 2. ERG is recruited to a subset of AR target genes and works together with EZH2 and HDAC1 and 2 in an integrated network to modulate the AR transcriptional outcome. EZH2-mediated methylation of lysine 27 of histone 3 (H3K27me) and histone deacetylation by HDAC1 and 2 across ERG-bound AR targets inhibit epithelial differentiation contributing to prostate carcinogenesis [66,67]. Similarly, ERG recruits PRTM5, which methylates AR on arginine 761. This attenuates AR recruitment to and AR-mediated regulation of genes normally expressed in differentiated prostate epithelium [68]. SETDB1 is another histone methyltransferase identified via a yeast two-hybrid screen to interact with ERG [69]. However, the relevance of this interaction for ERG biology is currently unknown but might be related to SETDB1-mediated gene silencing and maintenance of pluripotency [70,71].

## 4. ERG Fusions in Prostate Cancer

In addition to being overexpressed or rearranged in leukemia and Ewing’s sarcoma, ERG plays a central role in PCa tumorigenesis. ERG is weakly expressed in the normal prostate tissue compared to other ETS factors such as SPDEF and ERF that are important for normal prostate epithelium identity [72]. In 2005, seminal work by Tomlins and colleagues identified gene fusions between the AR-regulated gene *TMPRSS2* and *ERG* in a large subset of prostate cancer samples [73]. Although other ETS factors—such as *ETV1*, *ETV4* and *ETV5*—and AR targets—including *SLC45A3* and *NDRG1*—were reported to participate in gene fusions in PCa, *TMPRSS2-ERG* fusions are the most frequent, characterizing both initial and advanced stages of the disease [12,13,74,75,76]. *TMPRSS2* and *ERG* are about 3Mbp apart in the same orientation on chromosome 21. Through balanced or unbalanced (loss of the intervening sequence) translocation, the coding sequence of *ERG* is fused to the promoter and 5′ regulatory sequence of *TMPRSS2,* resulting in AR-mediated overexpression of ERG in the prostate cells. Interestingly, *TMPRSS2-ERG* fusions can bind the locus and regulate the expression of wild-type *ERG,* activating a feed-forward loop to maintain ERG expression in prostate cells [77]. Multiple *TMPRSS2-ERG* variants have been identified, some encoding the full-length ERG or a *TMPRSS2-ERG* fusion protein, while others produce an *N*-terminal truncated ERG, which retain the PNT and ETS domains that are essential for the transcription factor activity. Although the intronic DNA breakpoints vary, the most common fusion transcripts reported are the one between *TMPRSS2* exon 1 and *ERG* exon 4 and *TMPRSS2* exon 1 and *ERG* exon 5 [78,79,80].

AR itself appears to be necessary for *TMPRSS2-ERG* fusion formation. Indeed, activation of the AR signaling induces the three-dimensional proximity of the two genomic loci, which upon induction of DNA double strand breaks and aberrant repair, are fused together [81]. Several sources of DNA double strand breaks have been reported. Under experimental conditions, treatment with ionizing radiations and inflammation-induced oxidative stress lead to DNA breaks [82,83]. Furthermore, topoisomerase II beta (TOP2B) binds AR-regulated genes and the *TMPRSS2* and *ERG* fusion breakpoints and can trigger recombinogenic DNA double strand breaks [84]. To repair double strand breaks, microhomology-mediated non-homologous end joining (NHEJ) is activated contributing to de novo genomic rearrangements with *TMPRSS2-ERG* fusion formation [82]. The bromodomain protein BRD4 was also shown to facilitate DNA repair by promoting NHEJ and the expression of DNA repair genes, favoring *TMPRSS2* and *ERG* gene fusion [85]. On the contrary, the tumor suppressor NKX3.1 inhibits the juxtaposition mediated by AR of *TMPRSS2* and *ERG* loci and promotes homology-directed repair, thereby disfavoring *TMPRSS2-ERG* fusion formation [86,87].

*TMPRSS2-ERG* fusions are not detected in the normal prostate epithelium or in benign prostatic hyperplasia but are found in premalignant prostatic intraepithelial neoplasia (PIN) lesions, suggesting a role in the early stages of prostate cancer development [88,89,90,91,92,93]. Accordingly, tumor evolution analyses based on the genomic mutations detected in PCa patients support an early involvement of the *TMPRSS2-ERG* fusions in prostate tumorigenesis [94,95]. Furthermore, in vivo work revealed a causal role of ERG in initiating PCa. Although genetically engineered mouse models overexpressing ERG either showed no discernable phenotype [96,97,98] or developed a range of non-invasive phenotypes, including focal hyperplasia and PIN-like lesions [99,100,101,102], the levels of ERG expression seem to be critical for tumorigenesis. Indeed, Nguyen and colleagues identified and characterized age-dependent prostate tumors in transgenic mice expressing high levels of ERG [103]. Furthermore, a synergistic effect between ERG expression and perturbation of the PI3K pathway (e.g., PTEN loss or AKT activation) was reported in accelerating disease progression and promoting invasive and metastatic tumorigenic phenotypes in vivo [96,97,102,104]. Although considered an early event in prostate cancer, *ERG* gene fusions and overexpression can be detected in advanced disease, with only a modest decrease in frequency compared to primary PCa [12,13,105,106].

Controversies have emerged as to whether ERG activates or attenuates the AR signaling, leading to the hypothesis that ERG can promote the oncogenic functions of AR (e.g., promoting survival of the prostate cells) while inhibiting the tumor-suppressing ones (e.g., differentiation) [107]. In the context of PTEN loss, which suppresses AR and favors basal differentiation, ERG increases AR binding to chromatin and restores expression of the AR transcriptional output, thereby leading to the development of adenocarcinoma [102]. Moreover, biochemical assays showed that ERG directly interacts with AR and activates AR’s ability to bind DNA [108]. A recent study using established prostate cancer organoids derived from Pten^-/-^R26^ERG^ mice showed that *ERG* knockout dampens AR-dependent gene expression without altering AR binding to DNA or H3K27 acetylation at enhancers, but causing the loss of critical AR coregulators, such as NCOA3, and the basal transcriptional machinery [109]. To support AR signaling, ERG controls the expression of *AKR1C3*, an enzyme involved in the androgen biosynthetic pathway and the production of the AR ligands testosterone and dihydrotestosterone (DHT) [110]. Additionally, ERG promotes prostate luminal lineage by inhibiting the activity of a distal enhancer of *TP63*, a known master regulator of the basal prostate lineage, and through orchestrating chromatin interactions defining distinct transcriptional landscapes [111,112]. Accordingly, transcriptomic subtyping of prostate cancer patients’ samples showed enrichment of *ERG* overexpression in tumors with luminal features [113,114,115]. Of note, *TMPRSS2-ERG* indirectly upregulates the expression of *SOX9* by redirecting AR and activating an AR-regulated enhancer in the *SOX9* locus [116]. SOX9 is a critical downstream effector of ERG in *TMPRSS2-ERG*-positive cancer cells and induces neoplasia and tumor invasion when overexpressed in the murine prostate or cancer cells, respectively, similarly to ERG. Other works instead showed that ERG interacts with AR, co-occupies AR target genes and attenuates AR-mediated transcription by inducing a repressive epigenetic program activated by the H3K27 methyltransferase and subunit of PRC2 complex EZH2 and several HDACs, thus inhibiting AR-mediated lineage-specific differentiation [66,67,117]. Moreover, ERG recruits PRMT5, an arginine methyltransferase, to AR target genes to methylate and inhibit AR [68].

ERG overexpression in immortalized prostate epithelial and cancer cells increases the capacity of these cells to migrate and invade and controls the expression of genes involved in extracellular matrix remodeling, inflammation, migration and angiogenesis [96,99,100,102,118]. ERG regulates the expression of c-MYC, several metalloproteinases, chemokine receptors (e.g., CXCR4) and plasminogen activators (e.g., PLAU and PLAT) among others [96,99,118,119]. ERG also binds to and regulates transcription of several components of the WNT pathway, including ligands, the receptor FZD4 and the transcription factor LEF1, resulting in more active β-catenin and induction of epithelial-to-mesenchymal transition (EMT) in prostate cells [120,121,122]. EMT accompanied by reduced proliferation and cell cycle arrest in the G1 phase is also induced by *TMPRSS2-ERG* expression through TGF-β signaling with activation of the receptor ALK1, phosphorylation of p38 and upregulation of the transcription factors ZEB1 and ZEB2 [123,124,125].

Crosstalk between ERG activity and the NOTCH and NF-κB pathways was reported in PCa. Comparison of *TMPRSS2-ERG* positive and negative primary prostate tumors revealed the co-option of the prostate master transcription factors HOXB13 and FOXA1 and the activation of cis-regulatory elements present in NOTCH pathway-related genes in tumors overexpressing ERG [126]. The NOTCH pathway seems to be necessary for the viability and migration of cells overexpressing ERG, as shown by reanalysis of small hairpin RNA (shRNA) screen data from the Achille’s project and pharmacological inhibition [126,127]. Similarly, overexpression of *TMPRSS2-ERG* increases transcription of several NF-κB-related genes with concomitant phosphorylation at serine 536 of p65 [128]. Correlation between ERG expression and phospho-p65 was also observed in prostate cancer tissue microarray and expression of mutants mimicking the phosphorylated form promoted invasion and anchorage-independent colony formation of immortalized and tumorigenic prostatic cells [128,129]. Moreover, NF-κB inhibition decreased proliferation of the ERG fusion-positive PCa cell line VCaP [128].

## 5. Past Approaches to Target ERG Oncogenic Activity in Prostate Cancer

ERG fusions are found in about 40% of both primary and advanced PCa and define a distinct molecular subclass [12,13,105,130,131]. Loss-of-function mutations have been recently identified in factors that either target ERG for degradation or repress its functions [47,48,132], further highlighting the importance of the ERG pathway in this type of cancer. Accordingly, knockdown of ERG in models of advanced PCa endogenously bearing *ERG* fusions reduced cell growth, cell invasion and xenograft tumor growth, arguing that advanced tumors are dependent on ERG [66,99,133,134]. The high prevalence of ERG overexpression in PCa and the dependence of PCa cells on ERG activity for survival underscore the therapeutic potential of ERG. However, there are major challenges in developing therapeutics targeting an ETS transcription factor such as ERG. Transcription factors have been thus far considered ‘undruggable’ because of the lack of easily targetable binding pockets and the complex and often poorly understood regulation and function of individual transcription factor domains [135,136]. Furthermore, specificity in targeting ERG and its oncogenic functions is particularly important since ERG also has physiological roles and belongs to a large family of proteins with similar domains but opposite functions, with some being oncogenes, while others behave as tumor suppressors.

A first attempt to target ERG activity in PCa was prompted by the observation of the functional interaction between ERG and the DNA damage repair proteins PARP and DNA-PKc. Treatment of ERG overexpressing cells with the PARP inhibitor olaparib not only decreased ERG-mediated cell invasion and intravasation, but also inhibited growth in mouse xenograft models [65]. Mechanistically, ERG expression induced DNA double strand breaks, which further accumulated upon PARP inhibition. ERG-induced DNA damage was not due to alterations in homologous recombination efficiency and was independent of XRCC4-mediated NHEJ [65]. Another study instead reported the inhibition of DNA-PKcs functions by ERG with destabilization of critical NHEJ components, such as XCCR4, from chromatin [137]. Further in vitro work showed resistance to radiation of cells overexpressing ERG, which was reverted by inhibition of PARP [138,139]. Unfortunately, these compelling results did not hold in the clinical setting. Clinical trials failed to identify differences in the response rate of metastatic castration resistant prostate cancer (mCRPC) patients with or without *ERG* (and in general ETS) fusions to treatment with the PARP inhibitor veliparib and the androgen biosynthesis inhibitor abiraterone [140]. Moreover, ERG fusion status was not prognostic in patients with intermediate risk prostate cancer treated with radiation [141]. A recent in vitro study suggested that it might be worth re-assessing these results according to the PTEN, GSK3b phosphorylation and FBW7 status, since genotoxic therapies induce ERG degradation in prostate cancer cells only when these three proteins are functional [50].

HDACs were shown to be highly expressed and necessary for ERG activity in advanced prostate cancer models [66,67,117]. However, negative results were obtained from clinical trials testing the activity of HDAC inhibitors in CRPC patients [142,143,144]. 

In recent years, several small molecule inhibitors were developed to specifically inhibit ERG activity. The small molecule YK-4-279 was originally identified by surface plasmon resonance (SPR) and developed for Ewing’s sarcoma to block EWS-FLI1 binding to DHX9/RNA helicase A (RHA), an important cofactor for its oncogenic activity [145]. The efficacy of the treatment with YK-4-279 in inducing apoptosis and reducing tumor growth in Ewing’s sarcoma xenograft models and the high homology between FLI1 and other ETS members, in particular ERG, fostered the testing of YK-4-279 in PCa cells. YK-4-279 treatment inhibited ERG and ETV1 transcriptional activity and consequent cell invasion in VCaP and LNCaP cells, respectively, but had no effect on PC-3 cells overexpressing ETV4 [146]. Patient-derived xenograft models positive for ERG overexpression treated with YK-4-279 showed a heterogeneous response, with a significant decrease in cell proliferation and tumor volume and prostate-specific antigen (PSA) decline in one line and only partial response in another on three xenografts tested [147]. Nhili and colleagues identified the di-(thiophene-phenyl-amidine) compound DB1255 as an inhibitor of the ERG-DNA interaction. DB1255 was shown to specifically bind DNA at 5′-GGAA(g)TT-3′ sequences, which correspond to a portion of the defined ERG binding sites. This binding to the partial ERG motif was sufficient to hamper ERG-mediated activation of a luciferase reporter assay [148]. Compound VPC-18005 was developed by using a structure-based virtual screening approach followed by in vitro validation [149]. It binds the ETS domain pocket of ERG and disrupts its interaction with DNA, thereby inhibiting regulation of target genes, migration and invasion of ERG overexpressing cells in vitro and cell extravasation in zebrafish xenotransplantation experiments. The inhibitor WP1130 instead acts by destabilizing ERG [51]. By inhibiting the deubiquitinase USP9X, it favors ERG ubiquitination and degradation, leading to growth inhibition in PCa cells, ex vivo cultures and xenograft models overexpressing ERG. Similarly, a screen for small molecules in *TMPRSS2-ERG*-positive VCaP cells identified ERGi-USU as a compound able to reduce ERG protein levels and inhibit cellular growth in this cell line, while having few effects on ERG fusion-negative and endothelial cells [150,151]. ERGi-USU works by inhibiting RIOK2, a kinase required in ribosome biogenesis for the maturation of the 40S subunit. The link between this inhibition and the selective effect on ERG and ERG overexpressing cells still needs to be further investigated.

Alternative approaches to inhibit ERG activity included the use of RNA interference technology and peptides. Shao et al. designed and optimized two small interfering RNAs (siRNAs) targeting the most common *TMPRSS2-ERG* fusion isoforms and used liposomal nanovectors for their in vivo delivery. Upon treatment, tumor growth of VCaP xenograft models was reduced, although to a variable extent [152]. Wang and colleagues employed an iterative screening of a phage display random peptide library to isolate peptides interacting with ERG [134]. Two ERG inhibitory peptides were selected as specifically binding to ERG in the ETS domain, thereby blocking interaction of ERG with DNA and/or critical proteins, such as AR and DNA-PKcs. Importantly, these peptides suppressed tumor growth in vivo with no effects on ERG-mediated angiogenesis.

Despite all the aforementioned attempts developed over the past years to inhibit ERG activity, the clinical practice still lacks a therapeutic strategy to specifically target ERG.

## 6. Present Approaches to Exploit ERG in Prostate Cancer

The prognostic value of ERG expression in PCa still needs to be fully understood. This unclear significance might depend on different factors, including patient heterogeneity, the methods used to detect ERG positivity and the clinical outcome taken into consideration. Several studies assessed ERG potential as a prognostic biomarker in recent years (summarized in [153]). Of note, more consistent results are reported for cohorts of patients evaluating disease progression from precursor lesions or undergoing active surveillance. Patients with high-grade PIN and positive for ERG overexpression showed a higher frequency of PCa progression compared to ERG-negative patients [154]. In cohorts of patients managed expectantly, ERG positivity correlated with an increased risk of disease progression and was associated with a higher incidence of PCa-specific death [155,156]. Additionally, in agreement with in vitro and in vivo preclinical studies showing that ERG interacts with tubulin and alters microtubule dynamics leading to impaired docetaxel or cabazitaxel engagement, detection of *TMPRSS2-ERG* fusion in the blood of CRPC patients is predictive of resistance to taxanes [157,158]. No clear association was instead observed for ERG fusions and chromosome 8p or *BRCA2* loss, which were previously implicated in prostate cancer initiation and progression [12,13,159,160,161].

Ongoing clinical trials are further evaluating the prognostic and predictive value of ERG fusions in PCa patients at different stages of the disease or during treatment (e.g., trials evaluating the AR signaling inhibitors enzalutamide and apalutamide, PSMA theranostics, brachytherapy; see ClinicalTrials.gov for reference) and include the analysis of ERG fusion status both in primary and secondary outcome measures. Moreover, *TMPRSS2-ERG* fusion can serve as cancer-specific biomarker for early diagnosis of PCa and can be detected in urine samples via reverse transcription–polymerase chain reaction (RT-PCR) or a clinical-grade transcription-mediated amplification (TMA) assay [162,163,164,165,166,167]. This non-invasive diagnostic tool improves the performances of screening based on serum PSA levels to detect disease at early stages and to predict the presence of high-grade PCa on biopsy [168,169]. Although only useful in half of all PCa patients, the non-invasive detection of *TMPRSS2-ERG* fusion might reduce patient overtreatment.

## 7. Future Strategies to Target ERG-Positive Prostate Cancer

Although there is currently no specific therapy targeting ERG and its prognostic value is still unclear, there is interest in investigating new therapeutic avenues in the context of ERG-positive tumors as ERG fusion remains one the most frequently altered genes in PCa and its activity is essential for the viability of ERG-positive tumor cells. Table 1 summarizes the therapeutic strategies that might be implemented in the future to target ERG-positive PCa. 

**Figure 2:**
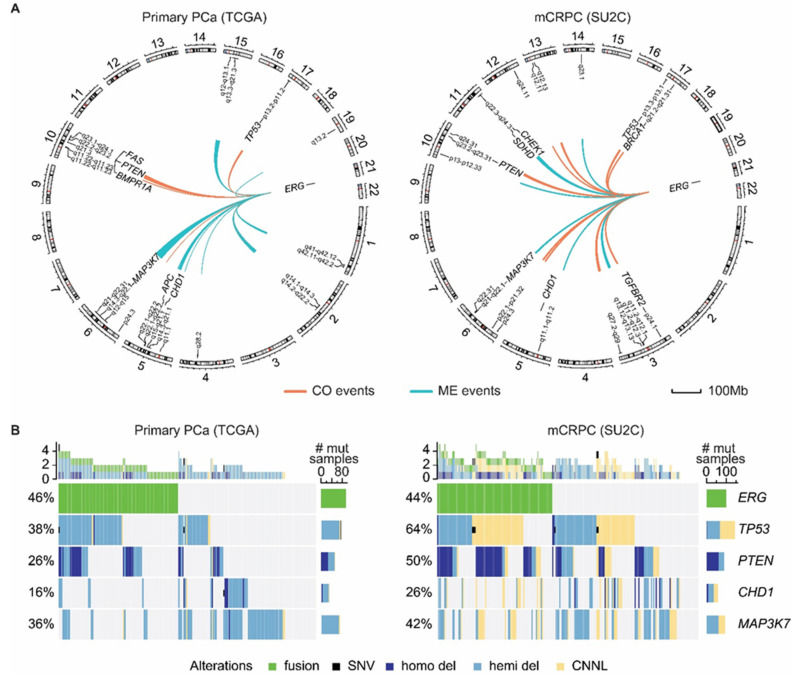
Mutually exclusive (ME) and co-occurrent (CO) genomic events with *TMPRSS2-ERG* fusion in PCa. (**A**) Circos plot of mutual exclusive and co-occurrent genomic events with *TMPRSS2-ERG* fusion in primary PCa (TCGA, **left**) and mCRPC (metastatic castration resistant PCa) (SU2C, **right**) datasets identified by FaMe (Fast Mutual Exclusivity) [185] upon in-house processing of genomic data at allele-specific levels. Single nucleotide variants (SNVs), hemizygous (Hemi) and homozygous (Homo) deletions, and copy number neutral loss (CNNL) were queried against tumor sample fusion annotation status from the original reports [12,13]. Tumor suppressors and oncogenes within significant genomic segments are highlighted. (**B**) Oncoprint of the data in A for genes with literature-based evidence.

Possible therapeutic hints might emerge by studying the underlying biology of mutually exclusive driver gene aberrations. The technological advances in next-generation sequencing have empowered the deep genomic and epigenomic characterization of localized and castration-resistant prostate tumor samples, revealing patterns of mutual exclusivity between *ERG* rearrangements and other key alterations (Figure 2 and [12,13]). Interestingly, the classification of prostate cancer samples based on transcriptomic characterization identifies similar patterns [114,178]. The causes for the detection of these patterns can be multiple, including (i) different cells of origin or initiating mechanisms leading to tumorigenesis, each characterized by specific mutations, (ii) functional redundancy of or abrogation of a selective advantage given by the second mutation, and (iii) synthetic lethality, when concomitant mutation of two genes results in cell death, while mutation of either gene alone is compatible with cell viability. SPOP mutation, *CHD1* deletion, *SPINK* overexpression, rearrangements involving other ETS members and deletion of a mega base-long region on the q arm of chromosome 6 are among the mutations mutually exclusive with *ERG* aberrations for which divergent tumorigenesis or functional redundancy are believed to underlie this pattern [12,105,131,179,180,181]. For instance, an independent mechanism driving PCa tumorigenesis was uncovered in PCa tumors overexpressing *SPINK1* with the activation of a gastrointestinal circuit driven by the transcription factors hepatocyte nuclear factor 4-gamma (HNF4G) and hepatocyte nuclear factor 1-alpha (HNF1A) [182]. Functional redundancy, instead, underlies the mutual exclusivity between SPOP mutations and *ERG* rearrangements—wild-type ERG protein is stabilized and the PI3K/mTOR pathway and the androgen receptor signaling are activated by mutant SPOP [47,48,183]. Yet, in vivo data failed to detect mutant SPOP-mediated stabilization of ERG [184]. Recently, Bernasocchi et al. further explored the functional connection between ERG and SPOP, identifying a synthetically sick interaction between these two proteins driven by the activation of incompatible pathways [170]. At the molecular levels, SPOP is upregulated by ERG, dampens AR signaling and sustains ERG activity through degradation of the histone reader ZMYND11. Conversely, mutant SPOP induces AR signaling and antagonizes ERG activity through stabilization of ZMYND11. From a therapeutic point of view, these results translate into a fine sensitivity of ERG-positive tumor cells to SPOP inhibition with a recently developed small molecule inhibitor [171]. Furthermore, treatment with supraphysiological androgen levels, a therapeutic strategy currently under clinical evaluation and showing antitumor activity in a subset of patients, might specifically benefit patients with ERG-positive tumors [172,173].

The continuous exploration of the vast collection of patient-derived tumor genomic data with the development of novel and more detailed analyses coupled with functional validation might lead to the identification of previously uncharacterized synthetic lethal interactions with potential therapeutic value. We recently implemented FaME, an algorithm for the Fast Mutual Exclusivity analysis of genomic aberrations [185] that can be applied to a variety of data. Its application to allele-specific information from the primary localized prostate cancer TCGA dataset (PRAD) [12,186] and from the mCRPC collection from the SU2C dataset [13] nominated mutually exclusive or co-occurrent aberrations with *ERG* fusion events. The analysis detected already-reported mutually exclusive and co-occurrent partners and further identified potential novel interactions (Figure 2). These data might shed light on ERG biology in PCa and potentially disclose novel therapeutic targets and strategies for the treatment of ERG-positive patients.

A promising route to exploit cancer-specific vulnerabilities is represented by therapies tailored to patient genomic characteristics. Intronic genomic breakpoints originated from the rearrangement of an AR-regulated gene with *ERG*—and in general from any somatic genomic aberration—represent cancer-specific sequences that could be used to selectively kill ERG-positive tumor cells. CRISPR-associated nucleases represent the state-of-the-art technology for targeting specific sequences in the genome. Although *ERG* knockout or expression inhibition via Cas9 and nuclease-null deactivated Cas9 (dCas9) functionalized with effector domains [187,188,189,190,191,192] might be difficult to exploit in the context of ERG rearrangements given their position mostly in non-coding regions and their long distance from the promoter, other strategies might be pursued. Recently, Chen and colleagues showed the feasibility of exploiting Cas9-mediated cleavage at breakpoints of gene fusions to induce tumors cell death [174]. They used Cas9D10A-based genome editing to introduce the suicide gene HSV1-tk, encoding the prodrug-converting enzyme herpes simplex virus type 1 thymidine kinase into the breakpoints of *TMEM135-CCDC67* and *MAN2A1-FER* fusions in human prostate cancer and hepatocellular carcinoma cells, respectively. HSV1-tk, but not the mammalian counterpart, phosphorylates the synthetic nucleoside homolog ganciclovir (prodrug), thereby enabling the block of DNA synthesis through elongation termination and the induction of cell death specifically in fusion and HSV1-tk positive tumor cells, while sparing fusion and HSV1-tk negative normal cells. Similar approaches might represent an innovative, genotype-specific approach for the personalized treatment of ERG-positive PCa patients. However, delivery and potential genotoxic effects remain two important issues to tackle for CRISPR-based therapies.

Small molecule inhibition might still be a pursuable strategy to target ERG-positive PCa. TK-216 is a derivative of the YK-4-279 inhibitor developed for EWS-FLI1 in Ewing’s sarcoma that is currently under evaluation in a phase II clinical trial (NCT02657005, [175]). Given the high similarity between FLI1 and ERG and the encouraging preliminary results obtained with YK-4-279 in PCa preclinical models overexpressing ERG (see Section 5), TK-216 might represent a promising therapeutic strategy for the treatment for PCa patients harboring tumors overexpressing ERG.

Computational approaches can be employed to reposition drugs to target ERG oncogenic activity. Gayvert et al. combined ENCODE ChIP-seq data with drug-induced expression profiles to pinpoint small molecules perturbing transcription factor activity. Dexamethasone, a glucocorticoid receptor (GR) agonist with anti-inflammatory activity, was identified as an inhibitor of ERG activity and decreased invasion and migration of ERG overexpressing cells in an AR- and GR-independent manner [176].

Proteolysis-targeting chimeras (PROTACs) have recently been developed to induce degradation of the protein of interest [193,194,195]. PROTACs are bifunctional chimeric molecules composed of a ligand that binds the target protein connected to a second ligand, engaging an E3 ubiquitin ligase. Treatment with a PROTAC results in the formation of a ternary complex, with the E3 ubiquitin ligase brought into proximity of the protein of interest, which is ubiquitinated and subsequently degraded through the proteasome. PROTACs have been developed to target AR [196,197,198,199,200,201], characterized by a ligand-binding domain that eases the design of the molecule. As already mentioned, ERG lacks such pocket that could interact with small molecules, rendering the development of PROTAC more difficult. To bypass this problem, DNA oligonucleotide can be linked to an E3 ligase ligand. As proof of principle, DNA double-strand hairpins including the binding sites of NF-κB and E2F were linked to the von Hippel–Lindau (VHL) ligand, leading to the degradation of the two transcription factors [202]. Similarly, 19-mer double-stranded oligonucleotides containing the ERG binding motif linked to different E3 ligase recruiting elements led to decreased ERG protein levels with concomitant downregulation of its target genes [177]. These preliminary results are compelling, yet the system might suffer from low specificity due to the fact that the binding motif of ERG can be recognized and bound also by other ETS members.

## 8. Conclusions

Since its discovery in 2005, a large body of evidence has emphasized the importance of ERG overexpression via fusion with androgen-regulated genes in PCa. Genomic, molecular and biochemical studies have shed light on the mechanisms inducing the fusion formation and the role of ERG in the early steps of the genesis of prostate cancer and in the advanced stages of the disease. Moreover, although the results of clinical studies on the prognostic value of *ERG* fusion detection in PCa patients remain unclear, its potential as diagnostic biomarker is evident as it is specific for prostate cancer and detectable in a non-invasive manner in the urine of patients. The availability of new research tools and model systems and the characterization of novel patient cohorts will help to further address ERG biology in PCa and other tumor types and to understand the differences between ERG physiological and pathogenic roles. This, together with the technological advances applied to the discovery of novel therapeutic strategies, will lay the groundwork for the development of inhibitors that specifically and efficiently target ERG fusion-positive tumors.

## Figures and Tables

**Table 1:** Future strategies to target ERG-positive PCa.

Therapeutic Strategy	References
SPOP inhibition	[170,171]
Treatment with supraphysiological androgen levels	[170,172,173]
CRISPR-based breakpoint specific insertion of suicide genes (e.g., HSV1-tk)	[174]
TK-216 (derivative of the YK-4-279 inhibitor developed for EWS-FLI1)	[145,146,147,175]
Dexamethasone (glucocorticoid receptor (GR) agonist with anti-inflammatory activity)	[176]
PROTACs (ERG binding motif linked to E3 ligase recruiting element)	[177]
